# Recovery of central memory and naive peripheral T cells in Follicular Lymphoma patients receiving rituximab-chemotherapy based regimen

**DOI:** 10.1038/s41598-019-50029-y

**Published:** 2019-09-17

**Authors:** B. Milcent, N. Josseaume, F. Petitprez, Q. Riller, S. Amorim, P. Loiseau, A. Toubert, P. Brice, C. Thieblemont, J.-L. Teillaud, S. Sibéril

**Affiliations:** 1 Cordeliers Research Center-Inserm UMR-S 1138, “Cancer, Immune Control and Escape” Laboratory, Paris, 75006 France; 2 Sorbonne Université, UMR-S 1138, Paris, 75006 France; 3 Paris Descartes-Paris 5 University, UMR-S 1138, Paris, 75006 France; 4 Ligue Nationale Contre le Cancer, Programme Cartes d’Identité des Tumeurs, Paris, 75014 France; 5 APHP, Saint-Louis Hospital, Hemato-oncology – Diderot University, Sorbonne Paris Cité, Paris, France; 6 Laboratoire d’Immunologie et Histocompatibilité, Hôpital Saint-Louis, Paris, France; 7 Inserm UMR-S 1160, Paris, France; 8 Institut Universitaire d’Hématologie, Université Paris Diderot, Paris, 7 France; 9 EA7324 Université Paris Descartes, Sorbonne Paris Cité, Paris, France; 10 Present Address: Laboratory “Immune Microenvironment and Biotherapy”, Sorbonne University UMRS1135, INSERM U.1135, Centre d’Immunologie et des Maladies Infectieuses (CIMI), Paris, France

**Keywords:** Adaptive immunity, Immunotherapy

## Abstract

Preclinical models and clinical studies have shown that anti-CD20-based treatment has multifaceted consequences on T-cell immunity. We have performed a prospective study of peripheral T-cell compartment in FL patients, all exhibiting high tumor burden and receiving rituximab-chemotherapy-based regimen (R-CHOP). Before treatment, FL patients harbor low amounts of peripheral naive T cells, but high levels of CD4^+^ T_EM_, CD4^+^ T_reg_ and CD8^+^ T_EMRA_ subsets and significant amounts of CD38^+^ HLA-DR^+^ activated T cells. A portion of these activated/differentiated T cells also expressed PD-1 and/or TIGIT immune checkpoints. Hierarchical clustering of phenotyping data revealed that 5/8 patients with only a partial response to R-CHOP induction therapy or with disease progression segregate into a group exhibiting a highly activated/differentiated T cell profile and a markedly low proportion of naive T cells before treatment. Rituximab-based therapy induced a shift of CD4^+^ and CD8^+^ T cells toward a central memory phenotype and of CD8^+^ T cells to a naive phenotype. In parallel, a decrease in the number of peripheral T cells expressing both PD-1 and TIGIT was detected. These observations suggest that the standard rituximab-based therapy partially reverts the profound alterations observed in T-cell subsets in FL patients, and that blood T-cell phenotyping could provide a better understanding of the mechanisms of rituximab-based treatment.

## Introduction

Follicular lymphoma (FL) is the second most common form of non-Hodgkin lymphoma (NHL). Its clinical course is highly variable and survival medians are 7–15 years depending on the studies. Follicular lymphoma management is characterized by a risk-adapted therapy based on the stage of the disease and the symptoms of the patients. For high tumor burden patients, treatment options could be either rituximab plus cyclophosphamide, vincristine and prednisone with (R-CHOP) or without (R-CVP) doxorubicin or other anthracyclines, or rituximab plus fludarabine for patients not eligible for anthracyclines, or rituximab plus bendamustine. Experimental therapies as well as allogeneic stem cell transplantation are rather considered for relapsed and more refractory disease^1^. The addition of the anti-CD20 monoclonal antibody (mAb) rituximab to chemotherapy has resulted in a higher rate of complete remission and improved survival^2^. In addition, rituximab as maintenance therapy after induction regimen improves progression-free survival (PFS) in high-tumor burden patients^3^. Nonetheless, about 15–20% of patients fail to respond to chemo-immunotherapy and die prematurely. New clinical-genetic predictors have been recently proposed to identify subgroups of patients at high risk of early failure of first-line immune-chemotherapy and disease progression (m7-FLIPI and POD24-PI)^4,5^. Huet *et al*. have also recently developed and validated a gene-expression profiling score applicable to formalin-fixed, paraffin-embedded tumor biopsies from patients with follicular lymphoma for predicting clinical outcome^6^. The gene signature defined in this study is characteristic of B-cell biology and tumor microenvironment. Gene expression profiling and molecular studies, as well as multiple immunochemistry and flow cytometry analyses of the microenvironment in lymph node biopsies have also highlighted the role of non-malignant immune cells and in particular tumor-infiltrating T cells (TIL) on outcomes of FL patients^7–19^. Only a few studies, however, have analyzed the relations between patient peripheral T-cell subsets and their clinical outcome^15,20–23^. The different therapies that have been used in cohorts of FL patients have complicated our understanding of the role of host adaptive immunity as many of them impacted both tumor and normal immune cells^24,25^. The infusion of anti-CD20 mAb affects T-cell compartments, as shown in both preclinical models and patients with inflammatory/autoimmune diseases or tumors^26,27^. Moreover, studies of mice engrafted with CD20^+^ tumor cells have demonstrated that anti-CD20 treatment, in addition to its antitumor effect based on innate immunity, has a major effect on tumor immune-surveillance through the development of long-term antitumor T-cell immunity^28–31^. Notably, we have previously shown that anti-CD20 treatment modifies the phenotype of CD4^+^ T cells by preventing the expansion of protumor CD4^+^ T_reg_ cells and inducing polarization towards a Th1 phenotype through the IFN-γ/IL-12 axis^29^. Anti-CD20-based treatment can have therefore multifaceted consequences on host adaptive immunity and affect T-cell subsets. An integrated phenotypic profiling of peripheral T-cell subsets in patients with follicular lymphoma before and during treatment could therefore provide a better understanding of the mechanisms of rituximab-based treatment.

We analyzed here the peripheral T-cell subset distribution in 33 FL patients with high tumor burden before any treatment. Thus, consistently with the PRIMA phase III study^3^, all patients received six cycles every 21 days of R-CHOP induction therapy. R-CHOP responder patients then received rituximab maintenance for two years. We report here a bias in peripheral T-cell subset distribution in 33 FL patients with high tumor burden before any treatment. Hierarchical clustering of multiparametric flow cytometry data revealed a group of patients characterized by a high proportion of both activated and PD-1/TIGIT expressing T cells. A significantly higher number of patients with partial response to rituximab-based therapy and with disease progression was observed in this group. These patients exhibited lower percentages of naive CD4^+^ T cells as compared to others FL patients. Moreover, the standard rituximab-based therapy in FL patients with high tumor burden induced the skewing of the effector memory phenotype towards a central memory and naive T-cell phenotype.

## Results

### Peripheral T-cell subsets in untreated FL patients differ from those in healthy donors

**Figure 1:**
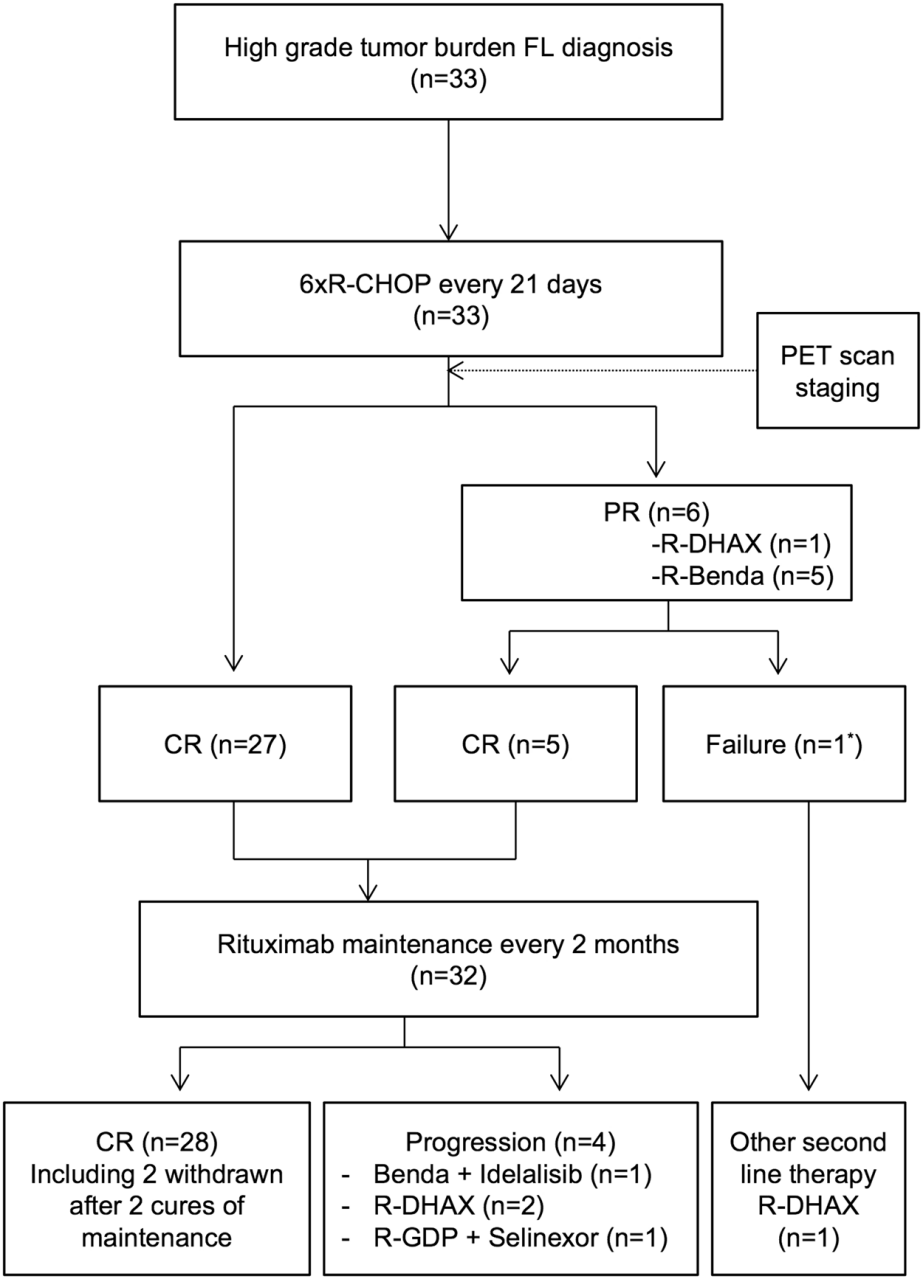
Flowchart of patients included in the study. The study included 33 patients diagnosed with high-tumor-burden Follicular Lymphoma (FL). The patients were treated with regimens based on rituximab and chemotherapy. CR = Complete Response. PR = Partial Response. PET = Positron Emission Tomography. R = rituximab. CHOP = cyclophosphamide, doxorubicine, vincristine, prednisolone. Benda = bendamustine. DHAX = dexamethasone, cytarabine, oxaliplatin. GDP = Gemcitabine, dexamethasone, cisplatin. ^*^This patient was one of the 5 patients who received R-Benda consolidation therapy following R-CHOP induction treatment.

33 high-tumor burden FL patients (grade I-III), with a majority of them (>80%) being in advanced stages III-IV of the disease, were enrolled in a prospective phenotypic study (Table 1). All patients were homogenously treated with an induction therapy containing rituximab and chemotherapy (R-CHOP) followed by either rituximab maintenance or chemotherapy-based consolidation regimens (Fig. 1 and Supplementary Table S1).

**Table 1:** Patients clinical characteristics before treatment.

Age at initiation of treatment (median ± S.D.) (*n* = 33)^*^	59 ± 10.77
**Sex (** ***n*** ** = 33)**	
Female	14/33 (42%)
Male	19/33 (58%)
**Grade (** ***n*** ** = 33)**	
Grade I-II	30/33 (90.9%)
Grade III	3/33 (9.1%)
**Ann Arbor stage (** ***n*** ** = 33)**	
I	1/33 (3.1%)
II	4/33 (12.1%)
III	11/33 (33.3%)
IV	17/33 (51.5%)
LDH elevated (*n* = 33)	7/33 (21.2%)
Serum Beta-2 microglobuline elevated (*n* = 15)	11/15 (73.3%)
Hemoglobin ≥ 12 g/dL (*n* = 33)	31/33 (94%)
Longer diameter of the largest involved node > 6 cm (*n* = 24)	13/24 (54.2%)
**FLIPI score** ^**a**^ **(** ***n*** ** = 33)**	
FLIPI ≤ 1	8/33 (24.2%)
FLIPI = 2	16/33 (48.5%)
FLIPI ≥ 3	9/33 (27.3%)

^*^n = Total number of patients for which the clinical data were available.

^a^Follicular Lymphoma International Prognostic Index (FLIPI) is based on a combined score of five parameters that include age (>60 *vs* ≤60 years), stage (III-IV *vs* I-II), anemia (hemoglobin <12 *vs* ≥12 dg/L), number of involved node areas (>4 *vs* ≤4) and serum LDH (elevated *vs* normal). FLIPI scores ≤1, 2, ≥3 classify patients into three groups with 10-year overall OS rates of 71%, 51% and 36%, respectively^51^.

**Figure 2:**
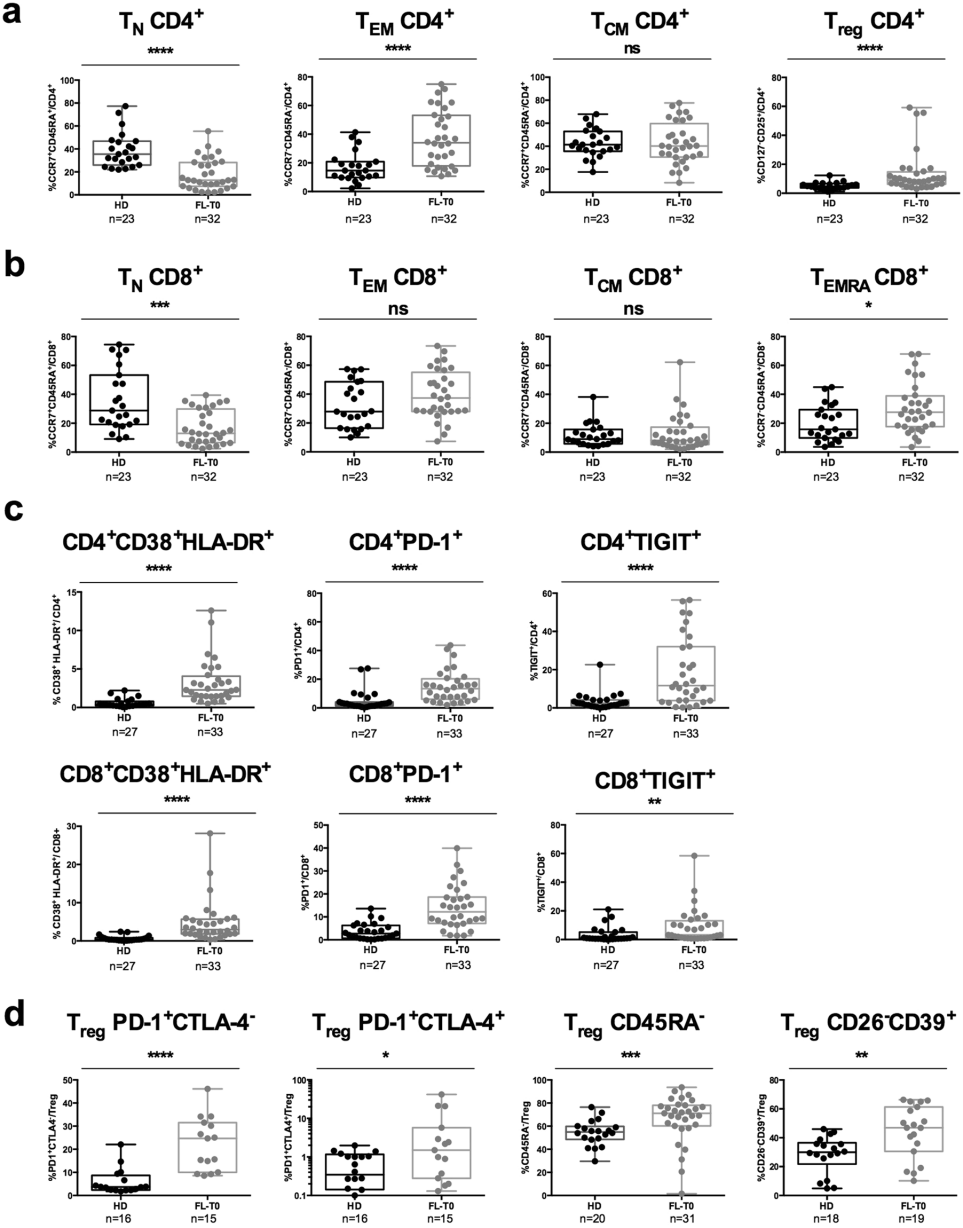
Analysis of peripheral T-cell subsets in FL patients before treatment. Box-and-whisker plots of flow cytometry data obtained from healthy donors (HD) and FL patients before treatment (FL-T0) blood samples. (**a**) Percentages of CCR7^+^CD45RA^+^ naive (T_N_), CCR7^−^CD45RA^−^ (T_EM_), CCR7^+^CD45RA^−^ (T_CM_) and CD127^−^CD25^+^ (T_reg_) CD4^+^ T cells. (**b)** Percentages of T_N_, T_EM_, T_CM_ and CCR7^−^CD45RA^+^ (T_EMRA_) CD8^+^ T cells. (**c)** Percentages of CD38^+^HLA-DR^+^, PD-1^+^ and TIGIT^+^ among CD4^+^ and CD8^+^ T cells. (**d**) Percentages of PD-1^+^CTLA-4^−^, PD-1^+^CTLA-4^+^, CD45RA^−^ and CD26^−^CD39^+^ among T_reg_. The number of samples that have been successfully processed are indicated below each panel. A Mann-Whitney test was performed for statistical analyses. *p < 0.05; **p < 0.01; ***p < 0.001; ****p < 0.0001; ns: not significant.

We first examined T-cell blood compartments of FL patients before any treatment. The percentages of CD4^+^ and CD8^+^ T cells did not differ between patients before treatment (FL-T0) and healthy donors (HD) (data not shown). However, when T-cell subsets were analyzed in detail, we observed that FL-T0 patients had a lower percentage of naive CD4^+^ T_N_ and CD8^+^ T cells than healthy donors did (Fig. 2a,b). Inversely, the percentages of CD4^+^ T_EM_, CD4^+^ T_reg_ (defined as CD25^+^CD127^−^) and of CD8^+^ T_EMRA_ were higher (Fig. 2a,b). Of note, the percentage of CD4^+^ T_EMRA_ was very low (<1%) (data not shown). Thus, subsets among this latter population were not further analyzed.

The activation status of blood T cells before any treatment was also compared to that of healthy donors (Fig. 2). The percentages of CD4^+^ and CD8^+^ T cells expressing CD38 and HLA-DR were significantly increased (Fig. 2c, left panels) due to an increase in the percentages of CD38^+^HLA-DR^+^ T_EM_, T_CM_, and T_EMRA_ cells (Supplementary Fig. S1). Moreover, the percentages of peripheral CD4^+^ and CD8^+^ T cells expressing PD-1 and TIGIT immune checkpoint molecules (ICP) were high (Fig. 2c middle and right panels), with up to 40–60% of T cells in some patients expressing these ICP. In contrast, the percentage of peripheral T cells expressing OX40, CD40L, GITR, 4-1BB, LAG3 and TIM3 was low, with a median value of stained cells below 1% in most cases (Supplementary Fig. S2). Higher percentages of CD45RA^−^ T_reg_ and CD26^−^ CD39^+^ T_reg_, as well as of PD-1^+^ or PD1^+^CTLA-4^+^ T_reg_, were also observed in FL patients suggesting an activation of this T-cell subset with an immunosuppressive capacity (Fig. 2d).

**Figure 3:**
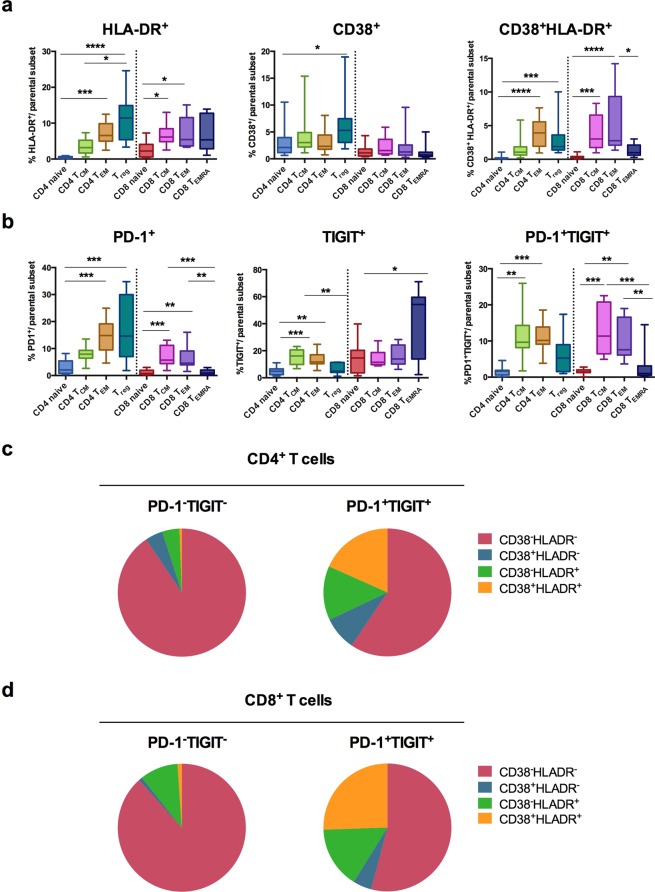
Activation status of peripheral T-cell subsets in FL patients before treatment. (**a**,**b**) Box-and-whisker plots of flow cytometry data obtained from blood samples of FL patients (*n* = 11) before treatment. Percentages of (**a**) HLA-DR^+^, CD38^+^, CD38^+^HLA-DR^+^ T cells and of (**b**) PD-1^+^, TIGIT^+^, and PD-1^+^TIGIT^+^ T cells among subsets of CD4^+^ and CD8^+^ T cells. (**c**,**d**) Pie chart of CD38/HLA-DR single-positive, double-positive and double-negative cells (slices represent the amount of each subset) among CD4^+^ (**c**) or CD8^+^ (**d**) T cells expressing PD-1 and TIGIT or not (indicated above each pie). A Kruskal-Wallis test followed by Dunn’s multiple comparison test was performed for statistical analyses of flow cytometry data. *p < 0.05; **p < 0.01; ***p < 0.001; ****p < 0.0001.

We then used an antibody panel (panel 4; Supplementary Table S2) to compare the expression and co-expression of CD38, HLA-DR, PD-1, and TIGIT in different T-cell subsets (Fig. 3). In line with the data reported above, T_CM_, T_EM_, and T_reg_ rather than naive T cells expressed CD38 and HLA-DR activation markers (Fig. 3a). Similarly, higher percentages of T_CM_ and T_EM_ expressed PD-1, TIGIT, or both molecules as compared to naive T cells (Fig. 3b). In addition, a large number of CD8^+^ T_EMRA_ cells expressed TIGIT (Fig. 3b, middle panel) and a high proportion of T_reg_ was PD-1^+^ (Fig. 3b, left panel). Moreover, the frequency of CD4^+^/CD8^+^ T cells expressing CD38 and/or HLA-DR among CD4^+^/CD8^+^ PD-1^+^TIGIT^+^ T cells was significantly higher than among CD4^+^/CD8^+^ PD-1^−^TIGIT^−^ T-cell subset (*p* = 0.0006 for both CD4^+^ and CD8^+^ T cells, using unpaired *t* tests) (Fig. 3c,d).

These data highlighted the presence in the blood of untreated FL patients of a substantial amount of CD4^+^ or CD8^+^ activated T cells also expressing PD-1 and TIGIT ICP. Of note, *in vitro* IFN-γ responses of PBMC from patients against CEFT peptides, derived from viruses commonly infecting large numbers of individuals (CMV, EBV, influenza) or from tetanus toxin, were similar to responses obtained with healthy donors (Supplementary Fig. S3).

Taken together, the decreased percentage of naive T cells associated with higher percentages of differentiated cells *(i*.*e*., T_EM_, T_CM_, T_EMRA_ and T_reg_ cells) expressing both activation markers and ICP molecules likely reflect an activation of T cells accompanying tumor development in FL patients prior to any treatment.

### High level of differentiated T cells expressing activation markers and PD-1/TIGIT ICP is related to the disease

Changes in peripheral T-cell compartments may be age-related as previously reported for naive and T_EMRA_ CD8^+^ T cells in other studies^32,33^. Thus, we performed correlation analysis between age and flow cytometry data in FL patients before treatment. The percentages of CD4^+^ and CD8^+^ T_N_, CD4^+^ T_EM_, CD8^+^ T_EMRA_, and of CD4^+^CD38^+^HLA-DR^+^ cells correlated with age (Supplementary Fig. S4). In contrast, age had no influence on the percentages of T_reg_ or of PD-1^+^ and TIGIT^+^ T cells. Also, no correlation was found between age and the percentage of CD4^+^ and CD8^+^ differentiated T cells (T_EM_, T_CM_, T_EMRA_) expressing both CD38 and HLA-DR (Supplementary Fig. S4). These results indicate that a part of peripheral T-cell phenotype alterations observed in untreated high-tumor burden patients is not related to age but to the disease.

### Patients with partial responses to R-CHOP or with early progression of the disease exhibit low percentages of naive CD4^+^ T cells before treatment

**Figure 4:**
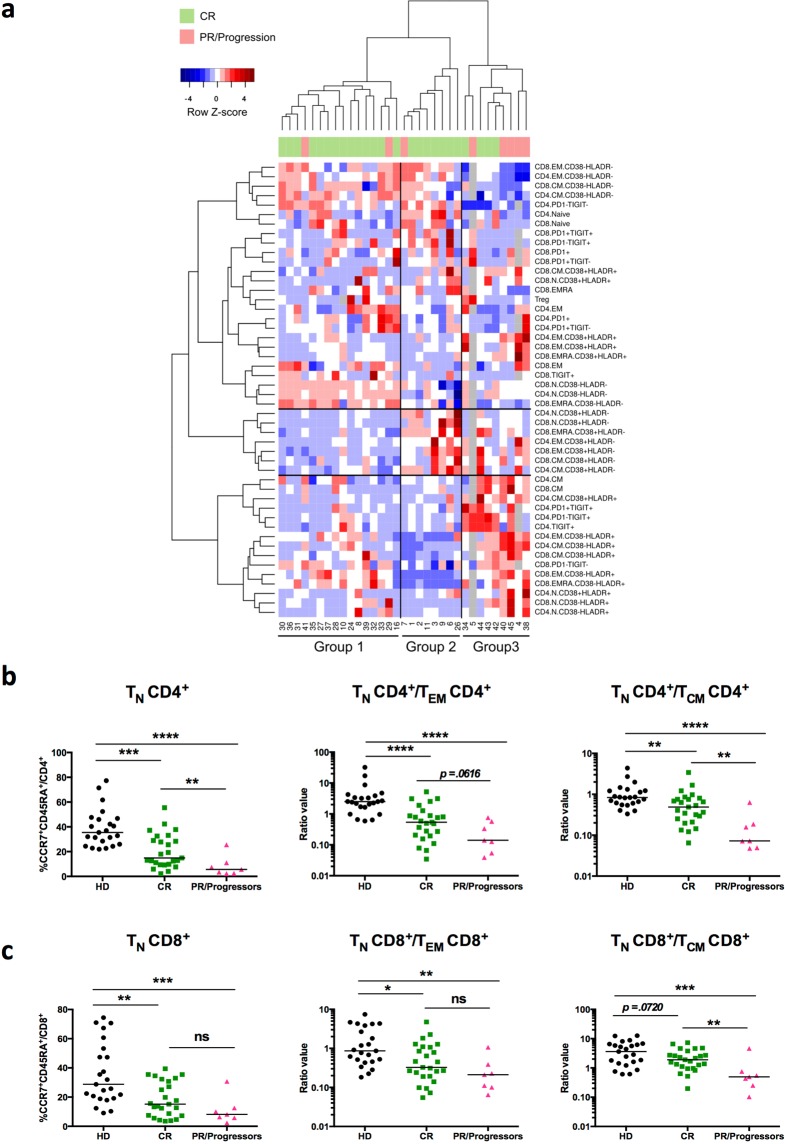
Heterogeneity of peripheral T-cell profiles in FL patients before treatment. (**a**) Hierarchical clustering of FL patients (*n* = 33) based on peripheral T-cell phenotypes before treatment. Phenotypes of analyzed T-cell subsets are indicated on the right. N indicates naive, CM central memory, EM effector memory, and EMRA effector memory RA^+^, CD4^+^ or CD8^+^ T cells. Patient numbers are indicated below the diagram. The heatmap is colored according to row Z-scores. Some of the values are missing in three patients (grey squares). Patient response to treatment is indicated below column dendrogram. Green = complete responders (CR); pink = partial responders (PR) and progressors. **(b**,**c)** Scatter plots of flow cytometry data obtained from pre-treatment blood samples of CR patients (*n* = 25) or PR/progressors patients (*n* = 7) or healthy donors (HD, *n* = 23). (**b**) Percentages of CCR7^+^CD45RA^+^ naive (T_N_) cells among CD4^+^ T cells and ratios of CD4^+^ T_N_/T_EM_ or T_N_/T_CM_ subsets. (**c**) Percentages of CCR7^+^CD45RA^+^ naive (T_N_) among CD8^+^ T cells and ratios of CD8^+^ T_N_/T_EM_ or T_N_/T_CM_ subsets. A Mann-Whitney test was performed for statistical analyses. *p < 0.05; **p < 0.01; ***p < 0.001; ****p < 0.0001; ns: not significant.

An unsupervised hierarchical clustering based on flow cytometry values obtained from the 33 patients was performed. We selected the data obtained with antibody panels #1, #2 and #3 (Supplementary Table S2) but not all the combinations from each panel were used. We excluded values (CD4^+^ T_EMRA_, OX40, CD40L, GITR, 4-1BB, LAG3 and TIM3 CD4^+^ or CD8^+^ T cells) for which the patients exhibited very low percentages (median value of stained cells below 1% in most cases). Three other values (% CD26^+^CD39^−^/T_reg_, % CD26^+^CD39^+^/T_reg_, % CD26^−^CD39^+^/T_reg_) for which we have the data from only 19 patients were also excluded. It demonstrated that, before treatment (FL-T0), a marked heterogeneity of the phenotypic profiles can be observed between patients (Fig. 4a). Three groups of patients with particular blood T-cell profiles could be identified. Group 1 (*n* = 16) is characterized by a low-level activation profile with a high frequency of naive CD38^−^HLA-DR^−^ CD4^+^ and CD8^+^ T cells and low proportions of CD4^+^ T cells expressing TIGIT. Moreover, in this group of patients, T_EM_, T_CM_ and T_EMRA_ subsets are mainly CD38^−^HLA-DR^−^. Inversely, patients from group 3 (*n* = 9) exhibited a high frequency of T_CM_, T_EM_, T_EMRA_ subsets expressing CD38 and/or HLA-DR activation markers. Patients of group 3 also exhibited high percentages of PD1^+^ and TIGIT^+^ CD4^+^ T cells. Finally, group 2 (*n* = 8) is characterized by intermediate activation profiles with high percentages of CD38^−^HLA-DR^−^ or CD38^+^HLA-DR^−^ T_CM_, T_EM_ and T_EMRA_ subsets and low percentages of T cells expressing PD-1 and TIGIT (Fig. 4a).

Among the 33 patients included in this study, 6 patients exhibited a partial response (PR) to the induction treatment (6 cycles of R-CHOP) and received a consolidation therapy based on chemotherapy combined to rituximab infusion. Tumor progression occurred later on for three of them. Moreover, 2/33 patients exhibited a disease progression during maintenance treatment (rituximab as a single agent) (Fig. 1 and Supplementary Table S1). Interestingly, group 3 that exhibits a differentiated and activated T-cell profile contained significantly more PR and progressor patients (5/8) than the two other groups characterized by T-cell profiles with low or intermediate activation markers (Fig. 4a) (Chi-squared test, *p* = 0.015). Moreover, before any treatment, PR and progressor patients exhibited significant lower frequencies of naive CD4^+^ T cells, and decreased CD4^+^ and CD8^+^ T_N_/T_CM_ ratios as compared to the others FL-patients and to healthy donors (Fig. 4b,c)

### Rituximab-based therapy induces profound changes in peripheral T-cell subsets

**Figure 5:**
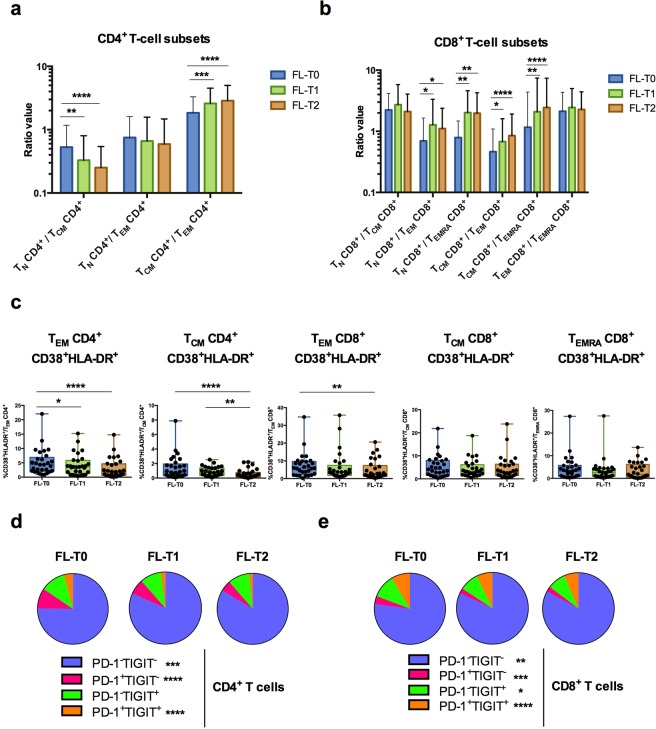
Changes in peripheral T-cell subsets in FL patients during therapy. Flow cytometry analysis of peripheral T-cell subsets in FL patients (*n* = 29) before (FL-T0) and during treatment (FL-T1; FL-T2). (**a**,**b**) Ratios of subsets of CD4^+^ (**a**) and CD8^+^ (**b**) T cells. (**c**) Box-and-whisker plots of percentages of CD38^+^ HLA-DR^+^ cells among T_EM_ and T_CM_ CD4^+^ T cells and T_EM_, T_CM_ and T_EMRA_ CD8^+^ T cells. (**d**,**e**) Pie chart of PD-1/TIGIT single-positive, double-positive and double-negative cells among CD4^+^ (**d**) or CD8^+^ (**e**) T cells (slices represent amount of each subset). Friedman test followed by Dunn’s multiple comparison test was performed for statistical analyses. *p < 0.05; **p < 0.01; ***p < 0.001; ****P < 0.0001; ns: not significant.

We then examined changes occurring in the peripheral T-cell compartments during rituximab-based therapy (FL-T1 and FL-T2). The percentage of CD4^+^ T_CM_ cells increased significantly at FL-T1 and FL-T2, whereas the percentages of T_N_, T_EM_ and T_reg_ CD4^+^ T cells decreased (Supplementary Fig. S5). For CD8^+^ T-cell subsets, the percentage of T_N_ cells and T_CM_ increased while the percentages of T_EM_ and T_EMRA_ CD8^+^ subsets decreased (Supplementary Fig. S5). Overall, these changes during treatment resulted in a decrease of the T_N_/T_CM_ ratio and an increase of the T_CM_/T_EM_ ratio in the CD4^+^ T-cell compartment (Fig. 5a). T_N_/T_EM_, T_N_/T_EMRA_, T_CM_/T_EM_, and T_CM_/T_EMRA_ ratios all increased in the CD8^+^ T-cell compartment (Fig. 5b). Taken together, these results revealed a shift throughout treatment from a T_EM_ phenotype toward a T_CM_ phenotype for both CD4^+^ and CD8^+^ T-cell compartments and also toward a naive phenotype for CD8^+^ T cells.

The activation status of peripheral T cells changed during treatment. The percentages of peripheral CD4^+^ T_EM_, CD4^+^T_CM_ and CD8^+^ T_EM_ cells expressing both HLA-DR and CD38, decreased during treatment (Fig. 5c). By contrast, the percentages of CD8^+^ T_CM_ CD38^+^HLA-DR^+^ and CD8^+^ T_EMRA_ CD38^+^HLA-DR^+^ remained stable during treatment (Fig. 5c).

The percentages of CD4^+^ and CD8^+^ T cells expressing TIGIT and/or PD-1 decreased noticeably during rituximab-based therapy (Fig. 5d,e). This reduction was closely linked to a diminution of PD-1^+^TIGIT^−^ and PD-1^+^TIGIT^+^ subsets among CD4^+^ T cells (Fig. 5d) and of PD-1^+^TIGIT^−^, PD-1^−^TIGIT^+^ and PD-1^+^TIGIT^+^ subsets for CD8^+^ T cells (Fig. 5e), as well to a marked increase in the number of PD-1^−^TIGIT^−^ CD4^+^ and CD8^+^ cells (Fig. 5d,e). Unsupervised hierarchical clustering based on flow cytometry at FL-T1 and FL-T2 did not allow defining groups of patients for their response to treatment (data not shown).

## Discussion

Studies using lymph node biopsies performed on retrospective cohorts of heterogeneous FL patient populations receiving various treatments have suggested that the amount of intra-tumor effector T cells expressing ICP, in particular TIM3, PD-1 and TIGIT, could have a prognostic value^22,23,34–36^. Only a few studies have investigated the phenotype of PBMC, in small-sized cohorts of untreated FL patients^20,21,23^. Modifications in the percentages of circulating naive helper CD4^+^ T cells and T_EMRA_ CD8^+^ T cells^21^, and in the amount of blood T_reg_^20^, have been demonstrated. However, the evolution of the peripheral T-cell subsets during FL treatment and the relationship between these subsets and the early response to R-CHOP have not yet been investigated. Thus, we have performed a longitudinal prospective study on peripheral blood from a small-sized cohort of similarly treated FL patients with high tumor burden, each receiving the same R-CHOP induction treatment followed by a maintenance treatment for most patients (25/33). Our study focused on the analysis of T-cell compartments, as preclinical data in mouse models as well as clinical investigations have suggested that T cells may play a major role in the control of tumor progression and in responses to antibody-based treatments^37^.

Here, we show that at the time of the first treatment decision, FL patients with a high tumor burden exhibit low percentage of naive CD4^+^ and CD8^+^ T cells. Taken together, the decreased percentage of naive T cells associated with higher percentages of T_EM_ expressing both activation markers and ICP molecules and of terminally differentiated TIGIT^+^ CD8^+^ T_EMRA_ (Figs 2 and 3) reflects a strong activation of T cells during tumor development in FL patients before any treatment. However, the median age of FL patients may be higher than that of healthy donors. Thus, we cannot exclude that a number of differences in peripheral T-cell compartments (including fewer naive T cells and more T_EMRA_ CD8^+^ T cells) observed in most untreated FL patients compared to healthy donors may be due, at least in part, to the senescence of the immune system, as previously observed for CD8^+^ T cells in other studies^32,33^. Nonetheless, it should be noted that in our study, the age has no influence on the percentages of T_reg_ or of PD-1^+^ and TIGIT^+^ T cells in FL patients. Whether FL patients with low tumor burden also exhibit the same pattern of phenotypic changes remains to be investigated.

Interestingly, despite PD-1 and TIGIT expression on effector T cells, *in vitro* IFN-γ responses to CEFT-derived peptides were not modified in PBMC of FL patients as compared to healthy donors (Supplementary Fig. S3). These results are consistent with another study showing that inhibitory receptors expression (including PD-1, CTLA-4 and TIM-3) on peripheral T cells is associated with their differentiation and activation, and does not necessarily correlate with reduced functionality^38^. Moreover, in a recent study, Josefsson *et al*., showed that tumors from NHL patients were enriched in CD4^+^ and CD8^+^ T_EM_ cells expressing both PD-1 and TIGIT and exhibiting reduced effector functions in immunosuppressive tumor microenvironment, but normal cytokine production upon *in vitro* culture in absence of their ligands^39^.

An unsupervised hierarchical clustering based on flow cytometry values led to the identification of three groups of patients with particular blood T-cell profiles (Fig. 4a). Group 3 exhibited a high frequency of T_CM_, T_EM_, and T_EMRA_ subsets expressing CD38 and/or HLA-DR activation markers, and high percentages of PD-1^+^ and TIGIT^+^ CD4^+^ T cells. This group contained significantly more PR and progressor patients (5/8) than the two other groups (Fig. 4a). Previous reports have demonstrated an association between the expression of activation markers in FL lymph nodes with a rapid transformation to aggressive disease and a non-responsiveness to rituximab therapy^8,40^. Moreover, we found that PR and progressors patients exhibited a significant lower percentage of naive CD4^+^ T cells, and decreased CD4^+^ and CD8^+^ T_N_/T_CM_ ratios as compared to CR patients and healthy donors groups (Fig. 4b,c). Interestingly, a recent study using mass cytometry for analysis of CD4^+^ T cell subsets in FL patient biopsies, has demonstrated that the frequency of intra-tumor naive T cells correlates with an improved patient survival^41^. These observations suggest that a careful investigation of T-cell profiles in the peripheral blood of FL patients with high tumor burden before treatment is of particular interest to decipher the mechanisms responsible for host failure to rituximab-based therapy.

Longitudinal flow cytometry phenotyping performed in FL patients also underlined changes in peripheral T-cell compartments during rituximab-based treatment. The therapy induced skewing of the T-cell compartment toward a CD4^+^ T_CM_ and CD8^+^ T_N_/T_CM_ profile (Fig. 5). Although the different roles of T_CM_ and T_EM_ in immunity to infection are well described, many questions remain about which type of memory T cells are most beneficial for the development and maintenance of antitumor immunity. A comparison of the antitumor protection achieved by adoptively transferred tumor-reactive T_CM_ versus T_EM_ CD8^+^ T cells revealed that the T_CM_ protective activity is superior to that of T_EM_, primarily due to the T_CM_ lymphoid homing properties that increase their exposure to tumor-associated antigens presented by DCs^42^. Finally, a recent study showed that chimeric antigen receptor-modified T cells (CAR T cells) directed against CD19 molecule derived from either the T_N_ or T_CM_ subsets conferred a superior antitumor activity in a xenograft mouse model of B-cell lymphoma^43^.

Part of the effects could be due to the CHOP (cyclophosphamide, vincristine, doxorubicin, prednisone) chemotherapy. It has been shown in humans or preclinical models that cyclophosphamide could deplete circulating T_reg_, promote Th1, Th17 memory responses and CTL-dependent immune responses^44^. However, some studies in B-cell depleted mice and in rituximab-treated patients with autoimmune diseases suggest that the impact of R-CHOP on T-cell compartment is a direct consequence of B-cell depletion and the absence of T-cell/B-cell cooperation. Notably, it has been reported that B cell depletion after treatment with rituximab in autoimmune disorders affects T cell differentiation and activation, including down-regulation of costimulatory molecules and activation markers (eg, CD44, CD40L, CD69, HLA-DR, ICOS) on CD4^+^ T cells, and increases in regulatory T cell numbers and suppressive functions^27^. Moreover, it has been shown that rituximab treatment could impact the naive/memory balance in T-cell subsets^45^. In naive mice treated with anti-mouse CD20 antibodies, short-term or chronic B-cell depletion disrupts CD4^+^ and CD8^+^ T cell homeostasis, in terms of naive, memory and effector T cell frequencies^26,46,47^. Also, immune responses to lymphocytic choriomeningitis virus (LCMV) infection are impaired, with a significant reduction in the number of IFN-γ and TNFα-producing T cells^46,47^.

In our study, changes in peripheral T-cell profiles was accompanied by a decrease of the percentages of peripheral T cells expressing the ICP PD-1 and TIGIT. Moreover, the amount of peripheral CD4^+^ T_EM_, CD4^+^ T_CM_ and CD8^+^ T_EM_ cells expressing both HLA-DR and CD38 also decreased during treatment (Fig. 5). This suggests that the blood T-cell profile observed in high tumor burden FL patients could be at least partially reverted by rituximab-based treatment, and that anti-tumor activity could be reinforced by an increase in the number of CD4^+^ T_CM_, CD8^+^ T_CM_ and CD8^+^ T_N_. Nevertheless, whether these changes have a long-term impact on the clinical outcome of high-tumor burden FL patients remains to be determined.

## Methods

### Ethical approval and ethical standards

This non-interventional study, which complies with the Declaration of Helsinki, was approved by the appropriate regional ethics committee (“Comités de Protection des Personnes”, Ile-de-France, France) regulated by the government institution “Agence Régionale de Santé” (Bobigny, France), by the government advisory board for data processing in health care (“Comité Consultatif sur le Traitement de l’Information en matière de Recherche dans le Domaine de la Santé”, Paris, France) (CCTIRS N°14.626), and by the French data protection authority (“Commission Nationale de l’Informatique et des Libertés”, Paris, France) (CNIL N°DR-2015-237). All patients and healthy donors provided informed written consent to participate in this research study. For anonymous healthy donors, written informed consents were obtained by the French state agency EFS (www.efs.sante.fr).

### Patients and study cohort

The 33 patients enrolled consecutively in the study from 2015 to 2016 were diagnosed with high tumor-burden FL and treated with a standard treatment regimen based on rituximab and chemotherapy (Hemato-oncology Department, Saint Louis hospital, Paris, France) (Table 1). High tumor-burden FL patients have at least one of the following features^3,48^: any nodal or extranodal tumor mass with a diameter of more than 7 cm; involvement of at least three nodal sites, each of which had a diameter of more than 3 cm; systemic symptoms; substantial splenic enlargement; serous effusion, orbital or epidural involvement, or ureteral compression (alone or in combination); and leukemia. All patients received an induction therapy (R-CHOP) consisting of six cycles every 21 days of rituximab combined to chemotherapy (cyclophosphamide, doxorubicine, vincristine, prednisolone). R-CHOP responder patients then received rituximab maintenance for two years. Flow chart of patient treatment is detailed in Fig. 1. Blood samples were collected before treatment (FL-T0) and at two different time points during treatment (FL-T1 at three months and FL-T2 at 8–12 months after initiation of treatment) as described in Supplementary Table S1. Blood samples from anonymized healthy donors (HD) were obtained from the French blood agency (EFS). Peripheral blood mononuclear cells (PBMC) were obtained from heparinized blood tubes by Hypaque-Ficoll centrifugation. HLA-A, -B, -C, -DRB1, -DQB1 and -DPB1-typing of patients were performed using the PCR-SSO (Polymerase Chain Reaction-Sequence Specific Oligoprobe) molecular method using the LABType SSO kits from One Lambda Inc. (Canoga).

### Flow cytometry

Cryopreserved PBMC samples from each patient from the different time points were tested in the same experiment, along with control samples from healthy donors. PBMC were stained with antibodies mixed in panels described in Supplementary Table S2. Data were acquired with a LSR Fortessa flow cytometer (BD Biosciences) and analyzed with Kaluza software (version 1.3, Beckman Coulter). Two different antibody panels were used to enable discrimination of CD3^+^ conventional T-cell subsets (Panel 1, Supplementary Table S2), including CD4^+^ and CD8^+^ T cells, CD45RA^+^CCR7^+^ naive T cells (T_N_), CD45RA^−^CCR7^+^ central memory T cells (T_CM_), CD45RA^−^CCR7^−^ effector memory T cells (T_EM_), CD45RA^+^CCR7^−^ effector memory RA cells (T_EMRA_), and of CD4^+^ CD127^−^CD25^+^ regulatory T cells (T_reg_) (Panel 2, Supplementary Table S2). The expression of CD27 and CD28 was also evaluated to confirm the phenotype of the different T-cell subsets^32,49^. A third panel also made it possible to evaluate the expression of different activating and inhibitory ICP (OX40, CD40L, GITR, 4-1BB, LAG3, TIM3, TIGIT, PD-1) to determine the activation status of peripheral CD4^+^ and CD8^+^ T cells (Panel 3, Supplementary Table S2). A fourth panel enables analysis of the co-expression of CD38 and HLA-DR, PD-1 and TIGIT on the different T-cell subsets (Panel 4, Supplementary Table S2).

### IFN-γ ELISPOT assays

1–2 × 10^5^ PBMC/well were incubated in IFN-γ ELISPOT plates (CTL) for 36 hours in serum-free medium with a single mixture of MHC-I and MHC-II restricted peptides derived from cytomegalovirus (CMV), Epstein-Barr virus (EBV), Influenza virus and tetanus toxin (CEFT peptides, 4 μg/mL each) (Panatecs). A positive threshold was set at ≥10 spot-forming-units (SFU) per 10^5^ cells after subtracting the background noise as previously described^50^. Each sample was tested in triplicate and the mean value was reported.

### Statistical analyses

Statistical evaluation of the results for flow cytometry and ELISPOT assays were performed using nonparametric unpaired (Kruskal-Wallis, Mann-Whitney), nonparametric paired (Friedman), and Spearman correlation tests (indicated in each figure legend). Kruskal-Wallis and Friedman tests were followed by *post-hoc* tests (Dunn’s multiple comparison test), performed with Prism software (version 5, Graphpad, San Diego, CA, USA). In one experiment, unpaired *t* tests with Bonferroni correction for multiple testing were used. Unsupervised hierarchical clustering was performed using R software (version 3.3.1), with Euclidian distance and Ward linkage criterion. A Chi-square test was used for comparison of the number of complete responders (CR) and partial responders (PR) patients among the groups defined by hierarchical clustering. *p* values for all statistical tests performed were considered significant when <0.05.

## Supplementary information

Supplementary information

## Data Availability

The datasets generated during and/or analysed during the current study are available from the corresponding author on reasonable request (sophie.siberil@sorbonne-universite.fr).
