# Supplementary material for: Recovery of central memory and naive peripheral T cells in Follicular Lymphoma patients receiving rituximab-chemotherapy based regimen
Source: Sci Rep. 2019 Sep 17;9:13471. doi: 10.1038/s41598-019-50029-y (PMC6748924; doi:10.1038/s41598-019-50029-y)
Supplement: Supplementary file 1 — Supplementary information [file 41598_2019_50029_MOESM1_ESM.pdf]

Recovery of central memory and naive peripheral T cells in Follicular Lymphoma patients receiving rituximab-chemotherapy based regimen

by

B. Milcent, N. Josseaume, F. Petitprez, Q. Riller, S. Amorim, P. Loiseau, A. Toubert, P. Brice, C. Thieblemont, J-L. Teillaud and S. Sibéril

Supplementary information

Supplementary Table S1. Patient clinical outcomes and time points of blood sample collection.

Supplementary Table S2. Antibody panels used in flow cytometry analyses of peripheral T-cell subsets.

Supplementary Figure S1. Percentages of peripheral T-cell subsets expressing CD38 and HLA-DR activation markers before treatment of FL patients.

Supplementary Figure S2. Percentages of peripheral T cells expressing immune checkpoints (ICP) in FL patients before and during rituximab-based therapy.

Supplementary Figure S3. *In vitro* IFN-γ responses to virus-derived peptides in FL patients.

Supplementary Figure S4. Age-dependence of peripheral T-cell phenotype in FL patients before treatment.

Supplementary Figure S5. Modifications of peripheral T-cell subsets during therapy.

Supplementary Table S1. Patient clinical outcomes and time points of blood sample collection.

Patient i.d.*	Timepoint T1 (FL-T1)	Response after induction treatment$	Timepoint T2 (FL-T2)	Clinical outcomes (Second line therapy)
001	After 5 cures of R-CHOP	CR	After 2 cures of R-maintenance	CR
002	After 5 cures of R-CHOP	CR	After 1 cure of R-maintenance	CR
003	After 5 cures of R-CHOP	CR	After 2 cures of R-maintenance	CR
004	After 5 cures of R-CHOP	CR	After 2 cures of R-maintenance	Progression (Bendamustine and Idelalisib)
005	After 5 cures of R-CHOP	PR	After 3 cures of R-Benda	Progression (R-DHAX and autograft)
006	After 5 cures of R-CHOP	CR	After 2 cures of R-maintenance	CR
007	After 5 cures of R-CHOP	CR	After 2 cures of R-maintenance	Progression (R-DHAX and autograft)
008	After 5 cures of R-CHOP	CR	After 2 cures of R-maintenance	CR
009†	After 5 cures of R-CHOP	CR	-	CR
010	After 5 cures of R-CHOP	CR	After 1 cure of R-maintenance	CR
011	After 5 cures of R-CHOP	CR	After 2 cures of R-maintenance	CR
016	After 5 cures of R-CHOP	CR	After 1 cure of R-maintenance	CR
024	After 5 cures of R-CHOP	CR	After 2 cures of R-maintenance	CR
026	After 5 cures of R-CHOP	CR	After 1 cure of R-maintenance	CR
027	After 5 cures of R-CHOP	CR	After 2 cures of R-maintenance	CR
028	After 5 cures of R-CHOP	CR	After 1 cure of R-maintenance	CR
029§	After 5 cures of R-CHOP	PR	At the end of induction therapy (6 cures of R-CHOP)§	CR
030	After 5 cures of R-CHOP	CR	After 2 cures of R-maintenance	CR
031†	After 5 cures of R-CHOP	CR	-	CR

032	After 5 cures of R-CHOP	CR	After 2 cures of R-maintenance	CR
033	After 5 cures of R-CHOP	CR	After 4 cures of R-maintenance	CR
034	After 5 cures of R-CHOP	CR	After 1 cure of R-maintenance	CR
035	After 5 cures of R-CHOP	CR	After 1 cure of R-maintenance	CR
036	After 5 cures of R-CHOP	CR	After 1 cure of R-maintenance	CR
037	After 5 cures of R-CHOP	CR	After 2 cures of R-maintenance	CR
038	After 5 cures of R-CHOP	PR	After 2 cures of R-Benda	CR
039	After 5 cures of R-CHOP	CR	After 1 cure of R-maintenance	CR
040	After 5 cures of R-CHOP	PR	After 2 cures of R-Benda and 4 cures R-DHAX	Progression (Autograft)
041	After 5 cures of R-CHOP	PR	After 2 cures of R-Benda and 2 cures of R-maintenance	CR
042	After 5 cures of R-CHOP	CR	After 3 cures of R-maintenance	CR
043	After 5 cures of R-CHOP	CR	After 2 cures of R-maintenance	CR
044	After 5 cures of R-CHOP	CR	After 4 cures of R-maintenance	CR
045	After 5 cures of R-CHOP	PR	After 6 cures of R-Benda	Progression (R-GDP and Selinexor)

CR = Complete Response. PR = Partial Response. R = rituximab. CHOP = cyclophosphamide, doxorubicine, vincristine, prednisolone. Benda = bendamustine. DHAX = dexamethasone, cytarabine, oxaliplatin. GDP = gemcitabine, dexamethasone, cisplatin. [*] Patients 012-015, 017-023 and 025 were not included in the present study. [$] All patients received an induction therapy (R-CHOP) consisting of 6 cycles every 21 days of rituximab combined to CHOP chemotherapy. For patients with CR after 6 cycles of R-CHOP, the induction therapy was followed by a maintenance regimen with cycles of rituximab as a single agent every three month for two years (R-maintenance). Patients with PR to induction therapy received a consolidation rituximab/chemotherapy regimen (R-DHAX or R-Benda) followed by R-maintenance for responder patients. † Severe side effects led to withdrawn after two cycles of R-maintenance. § After induction therapy, this patient received a consolidation therapy based on 4 cures of R-DHAX followed by R-maintenance.
Blood samples were collected from all patients before treatment (T0 also termed FL-T0).

Supplementary Table S2. Antibody panels used in flow cytometry analyses of peripheral T-cell subsets.

Fluorochrome	Panel 1	Panel 2	Panel 3	Panel 4
FITC	CD8 (RPA-T8[*])	CD26 (M-A261[*])	GITR (eBioAITR[$])	CD8 (RPA-T8[*])
PerCP-eF710	CD3 (SK7[*])	CTLA-4 (14D3[$])	TIGIT (MBSA43[$])	TIGIT (MBSA43[$])
PE	-	CD45RA (HI-100[*])	LAG3 (3DS223H[$])	CD3 (OKT3[$])
PE-CF594	CD45RA (2H4LDH11LDB9[*])	CD39 (TU66[*])	-	CD45RA (2H4LDH11LDB9[*])
PE-Cy7	CD28 (28.2[#])	CD25 (M-A251[*])	TIM3 (F38-2E2[$])	CD25 (M-A251[*])
APC	-	CD127 (HIR-7R-M21[*])	OX40 (ACT35[#])	CD127 (HIR-7R-M21[*])
AF700	-	-	CD8 (RPA-T8[*])	-
APC-Cy7	HLA-DR (G46-6[*])	-	CD40L (24-31[#])	HLA-DR (G46-6[*])
BV421	CCR7 (150503[*])	CD4 (OKT-4[$])	4-1BB (4B4-1[#])	CCR7 (150503[*])
Amcyan	Viability (Live/dead cell dye)[£]	Viability (Live/dead cell dye)[£]	Viability (Live/dead cell dye)[£]	Viability (Live/dead cell dye)[£]
BV605	CD38 (HB7[*])	CD3 (OKT3[$])	CD3 (OKT3[$])	CD38 (HB7[*])
BV650	CD4 (SK3[*])	-	CD4 (SK3[*])	CD4 (SK3[*])
BV786	CD27 (L128[*])	PD-1 (EH12.2H7[#])	-	PD-1 (EH12.2H7[#])

Antibody clones are indicated into brackets. Conjugated antibodies or fluorescent dyes were purchased from [*] BD Bioscience, [$] eBioscience, [#] Biolegend, [£] ThermoFisher Scientific.

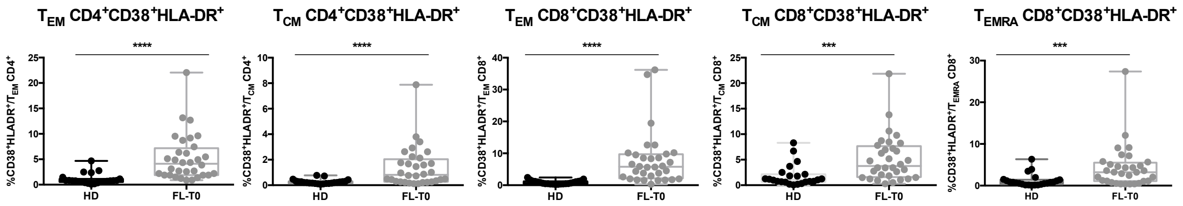

Supplementary Figure S1. Percentages of peripheral T-cell subsets expressing CD38 and HLA-DR activation markers before treatment of FL patients. Box-and-whisker plots of flow cytometry data obtained from blood samples of healthy donors (HD, n = 23) and FL patients before treatment (FL-T0, n = 32). T_{EM} and T_{CM} ($CD4^+$ and $CD8^+$) and T_{EMRA} ($CD8^+$) were analyzed. A Mann-Whitney test was performed for statistical analyses. *** $p < .001$; **** $p < .0001$.

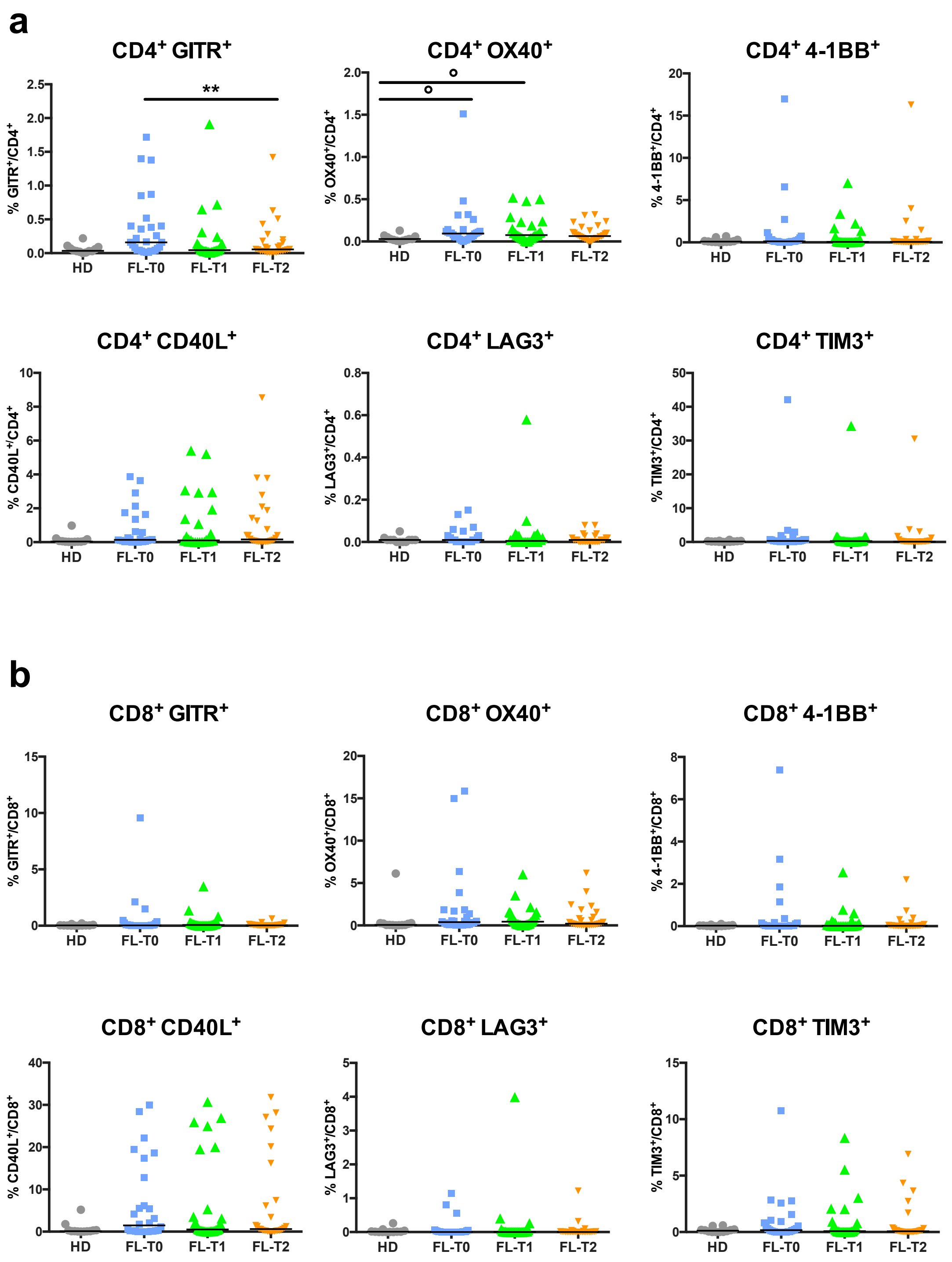

Supplementary Figure S2. Percentages of peripheral T cells expressing immune checkpoints (ICP) in FL patients before and during rituximab-based therapy. PBMC from healthy donors (HD) (n = 12) and from FL patients (n = 28) before treatment (FL-T0) and during therapy (FL-T1; FL-T2) were analyzed by flow cytometry. (**a**) Percentages of GITR$^+$, OX40$^+$, 4-1BB$^+$, CD40L$^+$, LAG3$^+$, TIM3$^+$ among CD4$^+$ T cells. (**b**) Percentages of GITR$^+$, OX40$^+$, 4-1BB$^+$, CD40L$^+$, LAG3$^+$, TIM3$^+$ among CD8$^+$ T cells. Friedman test (comparison between different time points in FL patients) and Kruskal-Wallis test (healthy donors *vs* patients) were performed for statistical analyses. Both tests were followed by Dunn's multiple comparison test. ° $p < .05$ (Kruskal-Wallis test); ** $p < .01$ (Friedman test).

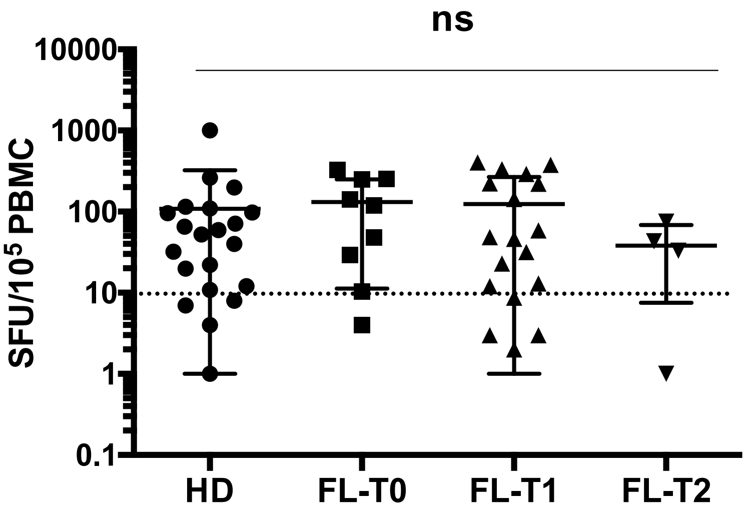

Supplementary Figure S3. *In vitro* IFN-γ responses to virus-derived peptides in FL patients. IFN-γ production in response to CEFT (CMV, EBV, influenza, tetanus toxin)-derived peptides by PBMC from healthy donors (HD; *n* = 21) and FL patients before (FL-T0, *n* = 9) and during (FL-T1, *n* = 18; FL-T2, *n* = 4) therapy, was assessed by ELISPOT assays. Results were expressed as spot-forming units (SFU) per 10^5 cells. A positive threshold was set at ≥ 10 spot-forming-units (SFU) per 10^5 cells after subtracting the background noise as previously described[50]. Each sample was tested in triplicate and the mean value was reported. A Kruskal-Wallis test followed by Dunn's multiple comparison test was performed for statistical analyses. ns: not significant.

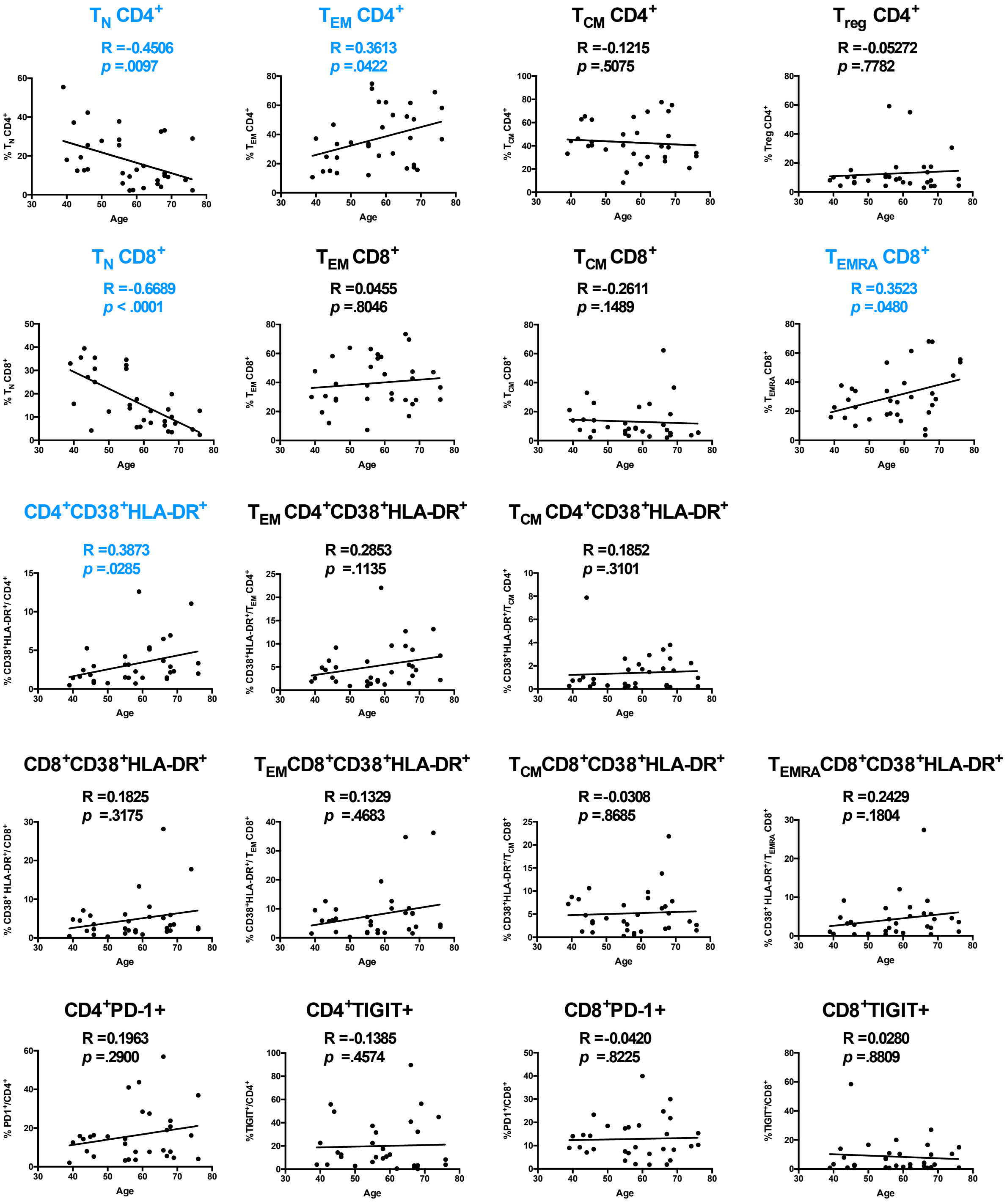

Supplementary Figure S4. Age-dependence of peripheral T-cell phenotype in FL patients before treatment. Spearman correlation tests were performed to analyze the relationship between the percentage of different T-cell subsets (indicated above each diagram) and age of the patients before treatment. R, correlation coefficient. Variables were considered as significantly correlated with $p < .05$ (in blue).

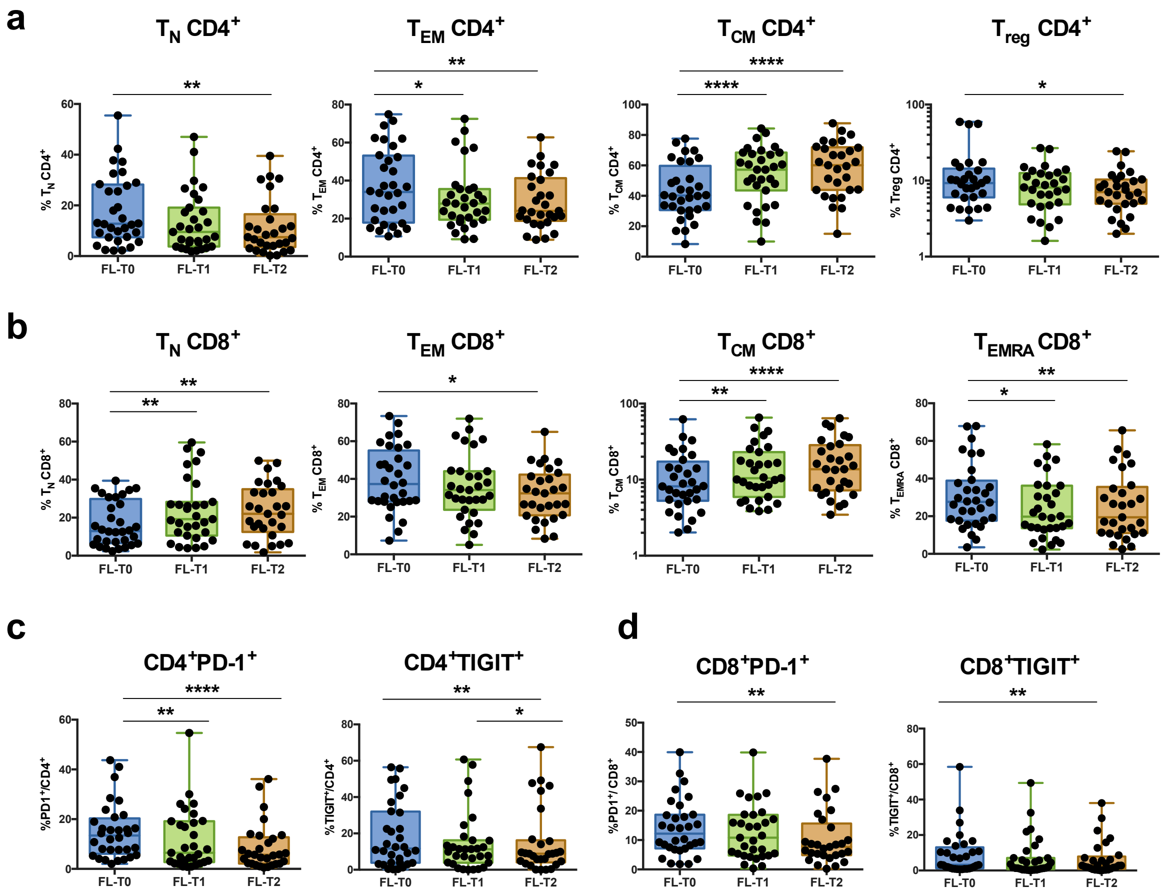

Supplementary Figure S5. Modifications of peripheral T-cell subsets during therapy. Box-and-whisker plots of flow cytometry data obtained from FL patients (n = 29) before (FL-T0) and during treatment (FL-T1; FL-T2). (**a**) Percentages of T_N, T_{EM}, T_{CM} and T_{reg} $CD4^+$ T cells. (**b**) Percentages of T_N, T_{EM}, T_{CM} and T_{EMRA} $CD8^+$ T cells. (**c**) Percentages of $CD4^+$ PD-1^+, $CD4^+$ $TIGIT^+$ T cells. (**d**) Percentages of $CD8^+$PD-1^+, $CD8^+TIGIT^+$ T cells. Friedman test followed by Dunn's multiple comparison test was performed for statistical analyses. * $p < .05$; ** $p < .01$; *** $p < .001$; **** $p < .0001$.
